# Pulmonary Delivery of Extracellular Vesicle-Encapsulated Dinaciclib as an Effective Lung Cancer Therapy

**DOI:** 10.3390/cancers14143550

**Published:** 2022-07-21

**Authors:** Qian Yuan, Kui Su, Shuyi Li, Xinyi Long, Lang Liu, Minghui Yang, Xin Yuan, Jianwu Sun, Junhua Hu, Qin Li, Yu Zhao, Zhengqiang Yuan

**Affiliations:** 1 School of Biomedical and Pharmaceutical Sciences, Guangdong University of Technology, Guangzhou 510006, China; 2112012086@mail2.gdut.edu.cn (Q.Y.); kuisu@mail2.gdut.edu.cn (K.S.); 2112012038@mail2.gdut.edu.cn (S.L.); 2112112050@mail2.gdut.edu.cn (X.L.); 2112112055@mail2.gdut.edu.cn (L.L.); 2112112049@mail2.gdut.edu.cn (M.Y.); 2112112060@mail2.gdut.edu.cn (X.Y.); 2112012059@mail2.gdut.edu.cn (J.S.); hu_jun_hua@163.com (J.H.); 2 Department of Food Science and Engineering, College of Food Science, Sichuan Agricultural University, Yaan 625014, China; liqin2020@sicau.edu.cn

**Keywords:** dinaciclib, EV engineering, pulmonary delivery, inhalation therapy, lung cancer

## Abstract

**Simple Summary:**

The clinical outcomes of lung cancer remain poor. The targeted delivery of treatment and the implementation of a method to overcome drug resistance are essential for the improvement of cancer therapy. The aim of our study was to assess the treatment effectiveness of engineered extracellular vesicles (EV) carrying both dinaciclib, a potent CDK inhibitor, and the proapoptotic factor TRAIL for a combinatorial lung cancer therapy. We showed that the engineered complexed EV agent, EV-T-Dina, was stable both in vitro and in vivo. Importantly, EV-T-Dina can overcome the drug-resistance of lung cancer cells, and when nebulized and administered by the pulmonary route, it demonstrated high efficacy and satisfactory safety for the treatment of lung cancers. The underlying mechanism for the synergistic killing of cancer cells by dinaciclib and TRAIL was associated with the concomitant downregulation of the anti-apoptotic factors cFLIP, MCL-1, and Survivin. Thus, the aerosolized EV-T-Dina potentially constitutes a novel and effective therapy for lung cancers.

**Abstract:**

The clinical outcomes of lung cancer remain poor, mainly due to the chemoresistance and low bioavailability of systemically delivered drugs. Therefore, novel therapeutic strategies are urgently needed. The TNF-related apoptosis-inducing ligand (TRAIL)-armed extracellular vesicle (EV-T) has proven to be highly synergistic for the killing of cancer cells with the potent cyclin-dependent kinase (CDK) inhibitor Dinaciclib (Dina). However, both optimal drug formulations and delivery strategies are yet to be established to facilitate the clinical application of the combination of EV-T and Dina. We hypothesize that Dina can be encapsulated into EV-T to produce a complexed formulation, designated EV-T-Dina, which can be nebulized for pulmonary delivery to treat lung cancer with potentially improved efficacy and safety. The prepared EV-T-Dina shows good stability both in vitro and in vivo and is very efficient at killing two highly TRAIL-resistant cancer lines. The ability to overcome TRAIL resistance is associated with the concomitant downregulation of the expression of cFLIP, MCL-1, and Survivin by Dina. The EV-T-Dina solution is nebulized for inhalation, showing unique deposition in animal lungs and importantly it demonstrates a significant suppression of the growth of orthotopic A549 tumors without any detectable adverse side events. In conclusion, the aerosolized EV-T-Dina constitutes a novel therapy, which is highly effective and safe for the treatment of lung cancers.

## 1. Introduction

As the leading cause of cancer death, lung cancer has the characteristics of high prevalence and frequent metastasis with a very limited efficacy of its first-line treatment. It was estimated that there were 2.2 million new lung cancer cases (11.4%) and 1.8 million deaths (18%) in 2020 worldwide [1]. Non-small cell lung cancer (NSCLC) is the major type of lung cancers accounting for around 85% of all cases.

Currently, the prognosis of lung cancer remains poor with an average five-year survival rate of only 16% despite many advances in diagnosis and treatment [2].The treatment failure is attributed to the low effectiveness of the first-line drugs [3], the tumors’ drug resistance [4], and an inappropriate drug delivery route [5]. Therefore, both a novel drug formulation design and more efficient approaches to drug administration are needed to improve the efficacy of lung cancer therapy.

Chemotherapy remains a primary approach for the treatment of lung cancers [6], and the systemic delivery of drugs through oral administration or injection is the common route. However, systemically administered drugs tend to be affected by the gastrointestinal (GI) acid environment [7], digestive enzyme activities, immune system-mediated clearance, and the hepatic first-pass effect [8], often resulting in significantly reduced drug bioavailability, a shortened half-life, and poor pharmacokinetics. To overcome these shortcomings, interdisciplinary technologies are being integrated for precision medication against cancers [9], such as nanotechnology [10], targeting modification, tissue engineering, novel formulation, chemical synthesis, and pharmacology.

The pulmonary delivery of aerosolized therapeutics may enable topical and more targeted lung cancer medication with maximized dosages and avoid the potential shortcomings of systemic administration [11]. Inhalational therapy may have special advantages over systemic treatments, such as a locally enriched drug deposition in lungs, the faster onset of anticancer effects, and a reduction in adverse side events.

Extracellular vesicles (EV) are an emerging nanoparticle vector for drug delivery [12,13], produced by most if not all cell types. As a natural nanoparticle vehicle responsible for the intercellular communication of cellular molecules such as mRNA, miRNA, and protein, EVs can be harnessed for the delivery of therapeutics by both pre-loading and post-loading strategies [14]. Mesenchymal stem cell (MSC)-derived EVs [15] are particularly ideal for the manufacture of anti-cancer nano-drugs [16], considering their innate merits such as their mass production by cells, lack of toxicity, low immunogenicity, antiapoptosis, antioxidation, antiinflammation, organ and tumor tropism, and versatile capacity for the loading of both hydrophilic and hydrophobic cargoes [17].

Previously, we successfully engineered MSCs to produce TNF-related apoptosis-inducing ligand (TRAIL)-armed EVs (EV-T) as an effective anticancer therapy [16]. Recently, we further demonstrated that EV-Ts could be combined with chemotherapies such as dinaciclib (Dina) and AZD5582, the latter being a small molecule antagonist of the inhibitors of apoptosis proteins (IAPs), to produce a strikingly enhanced therapeutic efficacy in lung cancer [18] or in hepatocarcinoma [19]. However, the optimal therapeutic formulation and delivery strategies are yet to be established to advance these therapies from bench studies to clinical application. In this study, we hypothesize that Dina can be encapsulated into EV-Ts to produce a complexed EV agent as a combination therapy, and the agent can be aerosolized for a pulmonary delivery to simultaneously improve its therapeutic efficacy and safety against lung cancers. The complexed EV therapeutics was evaluated to ascertain its potential to treat lung cancers both in vitro and in vivo.

## 2. Materials and Methods

### 2.1. Cell Culture

All cellular culture reagents were purchased from Gibco (Life Technologies, Gaithersburg, MD, USA) unless otherwise stated. Normal human umbilical cord (UC)-derived mesenchymal stem cells (UC-MSCs) and two NSCLC lines A549 and NCI-H23 (abbreviated to H23) were used in this study. A549 cells were luciferase (Luc) transduced. MSCs were purchased from the Shandong Yunzhou Biotechnology Co., Ltd., Zibo, China, and both A549 and H23 were obtained from the Fuheng Biology (Shanghai, China). A549 and H23 were grown in a DMEM medium containing high sugar (4.5 g/mL) and 10% fetal bovine serum (FBS); MSCs were cultured in the DMEM/F-12 medium supplemented with 10% FBS. Cells were cultured and maintained at 37 °C in a humidified atmosphere containing 5% CO_2_.

### 2.2. Preparation of TRAIL-Expressing Extracellular Vesicles (EV-Ts)

Previously established TRAIL-transduced human UC-MSCs (MSCflTs) [20] were used to produce TRAIL-expressing extracellular vesicles (EV-Ts). MSCflTs were first grown in complete DMEM/F12 medium of 10% FBS and cultured until reaching around 80% confluence, and were then incubated in an EV-producing medium that was supplemented with 10% EV-depleted FBS for 72 h. Subsequently, cell conditioned medium was collected; cleaned by a serial of low-speed centrifugations, including 10 min at 300× *g*, 10 min at 2000× *g*, and 30 min at 10,000× *g* to remove cells and cell debris; and then concentrated by using a centrifugal filter unit (Amicon^®^ Ultra-15,100 kDa MWCO, UFC910024, Merck KGaA, Darmstadt, Germany). Finally, the cleaned and concentrated supernatant was subjected to ultracentrifugation at 100,000× *g* for 2 h at 4 °C using the OptimaL-80 XP ultra-centrifuge equipment (Beckman Coulter, Brea, CA, USA) to precipitate EVs. The precipitated EV products were washed using filtered phosphate-buffered saline (PBS), resuspended in PBS, and aliquoted to store at −80 °C until use. Total EV proteins were determined using a BCA protein assay kit following the manufacturer’s instruction.

### 2.3. Characterization of Isolated EVs

A nanoparticle flow cytometer (nanoFCM, Xiamen, China) [21] was used to precisely determine the size distribution and particle concentration of isolated EVs. In addition, the morphology of EVs was examined using the transmission electron microscopy (TEM) according to previous description [5]. In brief, 5 µg of isolated EVs were absorbed on a TEM nickel grid, fixed, and negatively stained, followed by imaging with the Tecnai T12 electron microscope (FEI, Eindhoven, The Netherlands).

### 2.4. DiI Labeling and Cellular Tracking of EVs

EVs were labelled with the lipid membrane dye DiI (Sigma-Aldrich, Shanghai, China) for cellular uptake and tracking assay. EVs (60 µg) were first stained with 12 µM DiI for 25 min at RT in the dark. Then, labelled EVs were precipitated by ultracentrifugation at 100,000× *g* for 1.5 h to remove free dyes, and then resuspended in 100 kDa ultrafiltered PBS. A549 cells were first cultured in chamber slides for 24 h, and then co-incubated with DiI labeled EVs for 5 h and 11 h for endocytosis and intracellular tracking assay, respectively. Subsequently, treated A549 cells were washed by PBS, fixed with 4% paraformaldehyde and co-labelled with the cellular lysosome staining probe Lysotracker (Yeasen Biotech Co., Ltd., Shanghai, China), and finally examined and imaged by confocal microscopy (LSM800, Zeiss, Jena, Germany).

### 2.5. Detection of Bio-Distribution of DiR and DiR-Labelled EVs (EV-DiR)

EVs were labelled with the lipid membrane dye DiR (Sigma-Aldrich, Shanghai, China) to examine their in vivo biodistribution in lung tumor-bearing mice. EVs (200 µg) were first stained with 10 µM DiR for 30 min at RT in the dark. Then, labelled EVs were precipitated by ultracentrifugation at 100,000× *g* for 1.5 h to remove free dyes, resuspended in 100 kDa ultrafiltered PBS, and designated EV-DiR. Free DiR and EV-DiR were aerosolized and administered to lung tumor-bearing mice by inhalation, with 4 mice per group. Six hours post inhalation, mice were culled and their organs were isolated, including heart, liver, spleen, lung, kidney, and intestine for examination of DiR biodistribution by an in vivo imaging system (IVIS^®^ Lumina II Imaging System (excitation 745 nm, MILabs, Perkin Elmer, Shanghai, China) and recorded with a built-in CCD camera, respectively. The DiR fluorescence signal intensity was determined on the regions of interest and quantified using the Living Image software (MILabs, Perkin Elmer, Shanghai, China).

### 2.6. Preparation of Complexed EVs Carrying Both TRAIL and Dina

The potent cyclin-dependent kinase inhibitor Dinaciclib (SCH727965, Dina) was purchased from Selleck Chemicals (Shanghai, China) and dissolved in DMSO to prepare 10 mM stock solution. High-performance liquid chromatography (HPLC) was used to detect and measure Dina in solution using a C18 column (5 µm particle size, 250 mm × 4.6 mm; AAPPTec, Louisville, KY, USA) at 40 °C in isocratic mode with acetonitrile in water (95:5, *V*/*V*) as the mobile phase and at a flow rate of 0.8 mL/min. Dina elution was monitored using the UV-Visible DAD detector at 254 nm. A serial Dina concentration was measured to establish a detection standard curve, including 0.2-, 0.5-, 1.0-, 2.0-, 5.0-, and 10.0 µg/mL.

The dissolved Dina was encapsulated into EV-Ts to prepare the complexed agent EV-T-Dina. Three loading approaches were tested for Dina encapsulation in EV-Ts, i.e., co-incubation (mixing), via amphotericin B (AmB), or by sonication. The co-incubation was conducted to mix Dina and EV-T solution and incubate for 1 h at 4 °C. The AmB method consisted of adding 5% Amphotericin B (AmB, CAS1397–89-3, MedChemExpress, Shanghai, China) into the mixture of EV-Ts and Dina for co-incubation for 1 h at 4 °C. The sonication loading was performed by treating the Dina and EV-T mixture with sonication using a sonic dismembrator (Model 505). The sonication was set as the following: 20% amplitude, 6 cycles of 30 s on/off, with a 2 min cooling interval between each cycle. After sonication treatment, the sample was incubated for 1 h at 37 °C to restore the EV membrane integrity. EV-Ts with Dina loading treatment were subjected to ultracentrifugation at 100,000× *g* for 2 h to remove un-loaded Dina and precipitate EV-Ts again.

The encapsulated Dina was quantitated by measuring the UV absorption at 254 nm using an ultraviolet spectrophotometer. A standard curve of Dina/UV absorption was first established using the 254 nm absorbance as a function of Dina concentration (µg/mL). Afterwards, the EV-T-Dina solution (200 µL) was first evaporated at 50 °C using a vacuum concentrator, and then supplemented with acetonitrile (200 µL) and treated by sonication to disrupt EV membrane and completely release encapsulated Dina. Subsequently, the sonicated EV-T-Dina solution was precipitated by using the EV precipitating agent (ExoQuick-TC, System Biosciences, Beijing, China), and then Dina-containing supernatant was harvested for determination of Dina concentrations using the standard curve of Dina.

### 2.7. Preparation and Characterization of Aerosolized Agents

The therapeutic solution was converted to aerosol for inhalation therapy using a portable pressurized nebulizer (PARI BOY SX 085 G3055,PARI GmbH, Starnberg Germany). This nebulizer is designed to produce aerosolized droplets with a mean median diameter (MMD) of 2.2 µm, which is suitable for the inhalation-based treatment of lung cancer. An aerosol particle size analyzer (Spraytec STP2000, Malvern Instruments, Malvern, UK) was used to determine the nebulized particle size and size distribution. The injection cell was first inserted into the analyzer and warmed up for 4 h at 37 °C. Then, the air flow meter and the high-energy vacuum pump were connected, and the air flow rate was set as 30 L/min. Subsequently, the standard operating procedure (SOP) was set using the associated software (Spraytec software version 3.20, Malvern Instruments, Malvern, UK) as follows: test mode—fast mode; particle shading coefficient—1.33; dispersion medium—air. Next, the size analyzer was connected to the nebulizer and performed the size analysis of nebulized particles and droplets.

### 2.8. Evaluation of EV-T-Dina Stability In Vitro

Drug dialysis was carried out to assess and compare the stability of EV-T-Dina and free Dina. Both free Dina and EV-T-Dina were first dissolved in 2.0 mL of filtered PBS (pH 7.4) as 25.0 µg/mL in dialysis bags. Then, dialysis bags were incubated in 25 mL of PBS that was vortexed at 37 °C in a 50 mL flask in the dark. Every 1.0 mL of PBS was removed from the flask at various time points to measure released Dina by HPLC. The cumulative Dina release (*DR*) was calculated using the previously reported formula [22] as follows:

$$DR\%=\overset{n=t}{\underset{n=1}{\sum }}\frac{C_{n}V-C_{n-1}v}{m_{0}}\times 100$$

### 2.9. Evaluation of EV-T-Dina Stability In Vivo

To determine the in vivo stability of the drug [23], two groups of 6-week-old female BALB/c nude mice (5 in each group) were injected intraperitoneally with 100 mg/kg body weight of free Dina or Dina loaded in EV-T (EV-T-Dina), respectively. At 0.5 h, 1.0 h, 2.0 h, 4.0 h, and 8.0 h post injection, blood samples were collected through the tail vein and centrifuged at 2500× *g* for 15 min to remove coagulated blood cells and plasma proteins to produce serum, followed by measurement of serum Dina levels by HPLC.

### 2.10. Assessment of Cytotoxicity of Therapeutic Agents

The cytotoxicity of therapeutic agents was assessed by both cellular viability evaluation and apoptosis assay on A549-Luc, NCI-H23, and MSC cells following the previously established procedures [18]. In brief, the same number of cells were initially seeded in plate wells, cultured in plates and treated with vehicle or EV-T-Dina at various concentrations (0–40 nM Dina and 0–2.0 ng/mL TRAIL in combination) for 24 h, and then examined for their cellular viability and proliferation using the Cell Counting Kit-8 (CCK-8) (Dojindo, Kumamoto, Japan). Alternatively, cells were incubated with vehicle, EV-T-Dina, (2 ng/mL TRAIL combined with 20 nM Dina in EVs) and a TRAIL-neutralizing antibody (100 ng/mL, T3067, Sigma-Aldrich, Shanghai, China), alone or in combination for 24 h, followed by harvesting of both adherent and floating cells and labeling cells with FITC-Annexin V/propidium iodide (Bestbio, Shanghai, China) for apoptosis assessment by means of flow cytometry (FACS Calibur; Becton Dickinson, Franklin Lakes, NJ, USA). All experiments were performed in triplicates and repeated twice. The obtained results are presented as mean ± SEM (*n* = 3).

### 2.11. Detection of Protein Expression by Immunoblotting

Cellular or EV proteins of interest were detected by immunoblotting according to previous description [18]. In brief, 10 µg of total proteins extracted from each cellular or EV sample were separated by electrophoresis on 10% SDS-PAGE, transferred onto the PVDF membrane, and subsequently blocked and incubated with primary and secondary antibodies, respectively. Primary antibodies against the following proteins were used: human TRAIL (66756–1-Ig, Chicago, IL, USA) (dilution 1:1000), GAPDH (AF7021), TSG101 (DF8427, Affinity Biosciences, Cincinnati, OH, USA)) (dilution 1:2000), cFLIP (GTX113047, Houston, TX, USA) (dilution 1:2000), MCL-1 (Ab32087, Abcam, Cambridge, UK), and Survivin (Ab76424, Abcam, Cambridge, UK) (dilution 1:2000). Immunoblotting bands were quantitatively assessed by using the Image J software (National Institutes of Health, Bethesda, MA, USA) and normalized with the internal loading control protein GAPDH. The expression levels of proteins in treated samples were relative to control, for which the value was set to 1.

### 2.12. In Vivo Study

An orthotopic lung tumor model was established and used to evaluate the therapeutic efficacy of aerosolized EVs in BALB/c nude mice (female, 3–5 weeks old), which were obtained from an SPF Biotechnology Company (Huafukang Biotechnology Co. Ltd., Beijing, China). The experimental procedures and protocols were approved by the Animal Ethics Committee of South China University of Technology (Approval ID: 20211612526; Date: 25 June 2021). Animals were housed in pathogen-free animal facility with filtered air, autoclaved water, and food available all the time.

Luciferase-expressing A549 cells (A549-Luc) were resuspended in PBS (5 × 10^7^ cells/mL) and used for the preparation of an A549-Luc-containing Matrigel matrix (4.35 mg/mL) (BD Biosciences, San Jose, CA, USA). Mice were first anesthetized and placed in the right lateral decubitus position. Then, 1 mL syringes with 29 gauge needles were used to percutaneously implant A549-Luc cells in Matrigel (5 × 10^6^ cells/animal) into the right lateral thorax at the lateral dorsal axillary line, which is about 1.5 cm above the lower rib line and just below the inferior border of the scapula [24]. On day 21 post A549-Luc implantation, luciferase bioluminescence was measured to confirm the adequate development of A549 xenograft lung cancers by using an in vivo imaging system (IVIS^®^ Lumina II Imaging System (MILabs, Perkin Elmer, Shanghai, China). Then, 20 mice were randomly divided into four experimental groups with 5 animals per group: vehicle control (Ctrl), Dina treatment (Dina), EV-T treatment (EV-T), and EV-T-Dina treatment (EV-T-Dina). Aerosol delivery of therapeutics was performed for approximately 30 min/day for 7 times in total for each mouse—with an interval of 2 days between treatments—using a portable nebulizer (PARI, PARI BOY SX 085 G3055, PARI GmbH, Starnberg Germany) [25]. The doses were 0.2 M sucrose for Ctrl, 20 µg Dina/daily/mouse for Dina, 20 ng EV-T TRAIL/daily/mouse for EV-T, and 20 µg Dina encapsulated in 20 ng EV-T TRAIL/daily/mouse for EV-T-Dina group. Two days after the cycle of 7 inhalation treatments, animals were first imaged for bioluminescence, then culled to collect organs for immuno-histochemistry analyses. Animal body weight and general condition were monitored and recorded every 3 days.

### 2.13. Immuno-Histochemistry (IHC) Analyses

Twenty-four hours after the last treatment, mice were sacrificed and organs including heart, liver, spleen, kidney, and lung were resected for immuno-histochemistry (IHC) analyses following the procedures described previously [18]. TUNEL assay was performed for organs including the heart, liver, spleen, and kidney to examine therapy’s safety. Lung tumors were examined for cellular proliferation marker Ki67 and apoptosis indicator, the cleaved/activated caspase-3 (C-CASP-3), by IHC staining using specific Ki67 and C-CASP-3 antibodies (Cell Signaling Technology, MS, USA), respectively.

### 2.14. Statistical Analysis

The software GraphPad Prism 8.0 was used to analyze data (GraphPad Software Inc., La Jolla, CA, USA). Significant differences between two groups were analyzed using Student’s *t*-test, and comparison among multi-groups was performed using one-way ANOVA/Bonferroni multiple-comparison post hoc correction. Significant probability values were indicated as * *p* < 0.05, ** *p* < 0.01, and *** *p* < 0.001.

## 3. Results

### 3.1. Engineering Extracellular Vesicles to Deliver Both TRAIL and Dina

Dina and TRAIL were previously shown to synergize to induce apoptosis in cancer cells [18]. Therefore, we tried to load both TRAIL and Dina in EVs to prepare combined and aerosolized therapeutics for the potentially improved efficacy of the therapy.

**Figure 1:**
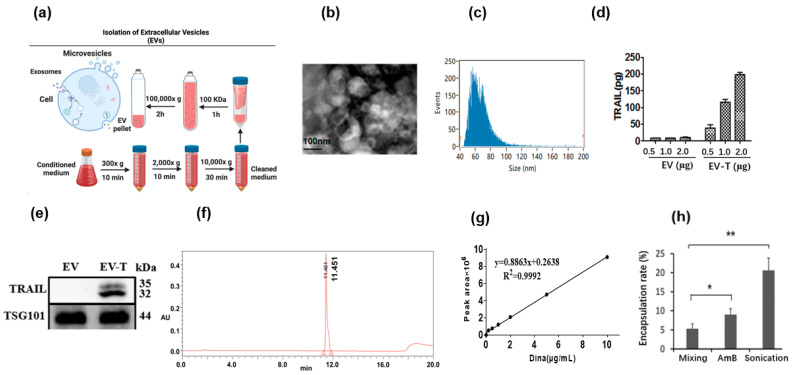
Isolation of TRAIL-expressing extracellular vesicles (EV-T) and post-loading of Dinaciclib (Dina) into EV-T to prepare the complexed therapeutics (EV-T-Dina). (**a**) Isolation of extracellular vesicles (EVs) by ultracentrifugation; (**b**) Examination of isolated EV-Ts by transmission electron microscopy (TEM); (**c**) EV-T analysis by high sensitivity flow cytometry; (**d**) Assessment of TRAIL expression in EV-Ts by a commercial human TRAIL-specific ELISA kit; (**e**) Expressional detection of TRAIL and the EV biomarker TSG101 in EVs and EV-Ts by immunoblotting. Uncropped blots are available in Figure S2; (**f**) Detection of Dina by high-performance liquid chromatography (HPLC); (**g**) Establishment of a linear regression curve and function for the detection of Dina; (**h**) Determination and comparison of Dina encapsulation rates by three loading methods of mixing, amphotericin B (AmB), and sonication. Values are means ± SD (*n* = 3), * *p* < 0.05, ** *p* < 0.01, by one-way ANOVA/Bonferroni multiple-comparison post hoc test.

TRAIL has been shown to be secreted via EVs in a nanosomal form (EV-T) by TRAIL-overexpressing cells [16,18]. So, the previously established TRAIL-transduced mesenchymal stem cell line (MSCTRAIL) [16] was used to produce EV-Ts in this study. A cell-conditioned medium was collected and first cleaned by sequential low-speed centrifugations to remove the cells and cellular debris, and then concentrated using a 100 kDa centrifugal filter unit, followed by ultracentrifugation for 2 h at 100,000× *g* to precipitate the EVs (Figure 1a). The isolated EVs were shown to be membranous vesicles of sizes ranging around 45–110 nm in diameter via transmission electron microscopy (TEM) (Figure 1b), with a mean size of 67.15 nm in diameter and a concentration of 3.91 × 10^10^ particles/mL measured by nanoparticle flow cytometry (Figure 1c). The TRAIL expression was assessed in the isolated EVs by using a TRAIL-specific ELISA, revealing 125.2 ± 15.2 pg of TRAIL per 1 μg of EV-Ts, while the EVs from the control MSC cells exhibited no detectable TRAIL expression (Figure 1d). The TRAIL expression was further confirmed in EV-Ts but not in EVs by immunoblotting, showing two bands of ~35 kDa and ~32 kDa, respectively (Figure 1e).

A high-performance liquid chromatography (HPLC) analysis was performed to isolate Dina and the built-in UV spectrometer was used to measure Dina concentration, showing a detection peak at 11.451 min (Figure 1f). The detection peak area is proportional to the Dina concentration with a linear regression function of y = 0.8863x + 0.2638, (R^2^ = 0.9992) (Figure 1g).

To prepare the EV-based aerosol for lung cancer treatment, we first needed to encapsulate Dina into the EV-Ts to produce complexed EVs that carry both TRAIL and Dina, a composite which has been designated EV-T-Dina. Three loading methods were tested for Dina’s encapsulation into the EV-Ts, which were mixing, sonication, and amphotericin B mediated approach. As shown in Figure 1h, the simple mixing of Dina and EV-T resulted in a Dina loading rate of 5.28 ± 1.25%, and the use of amphotericin B significantly improved the loading rate to 8.96 ± 1.55%. By contrast, the sonication treatment obtained the highest encapsulation rate of 20.56 ± 3.28%. The sonication-mediated Dina loading was thus determined as the optimal strategy for loading Dina into the EV-Ts to prepare the complexed EV-T-Dina agent in this study.

Free Dina in solution can be directly measured by using a UV spectrometer. However, the encapsulated Dina needs to be released from the EVs by sonication in acetonitrile for an accurate quantification. The loading rates via sonication were tested for different combinations of 100 µg/mL of EV-Ts with various Dina concentrations (1.0–100 µM), and the encapsulated Dina contents appeared proportional to the initial Dina concentrations. However, the Dina loading rates remained around 20.6% for various Dina loading concentrations. Therefore, for various Dina loading concentrations (1.0–100 µM, corresponding to ~0.4–40.0 µg/mL), the encapsulated Dina contents were measured and approximately calculated as 0.824–82.4 ng/µg EV-Ts.

### 3.2. EV Encapsulation Strikingly Improves Dina Stability Both In Vitro and In Vivo

**Figure 2:**
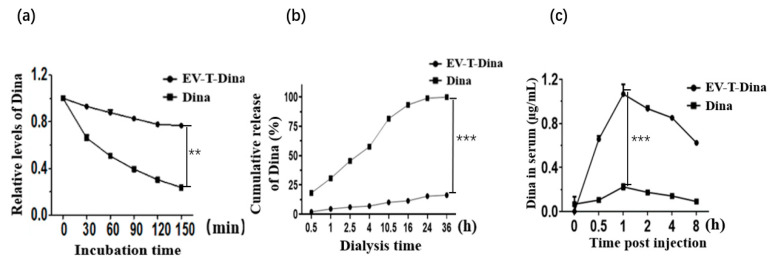
Assessment of stability of encapsulated Dina both in vitro and in vivo. (**a**). Examination of stability of encapsulated Dina in vitro. Free Dina (39.6 µg/mL, 100 µM) and EV-T-Dina (equivalently 7.92 µg/mL encapsulated Dina, 20 µM) were incubated at 37 °C for 0–150 min, followed by HPLC measurement of Dina. The remaining Dina was quantified and presented as relative to initial Dina concentration, for which the value was set as 1.0. (**b**). Analysis of Dina release in dialysis assay. Free Dina (30 µg/mL, 2 mL) and EV-T-Dina (equivalently 7.92 µg/mL encapsulated Dina, 2 mL) were dialyzed at room temperature (RT) in PBS (25 mL) in a vortexed flask in the dark, and 1.0 mL of sample was taken each time from the flask to measure the cumulative release of Dina at indicated time points. Each time point was repeated 3 times. (**c**). Determination of Dina and EV-T-Dina stability in vivo. BALB/c nude mice were injected intraperitoneally with free Dina at the dose of 100 mg /kg or EV-T-Dina at the same dose (corresponding to the encapsulated Dina 8.24 mg/kg), respectively, and blood sample was detected for Dina retention after 0.5–8.0 h. Three mice were tested for each group. All values are means ± SD (*n* = 3), ** *p*< 0.01, *** *p*< 0.001, by Student’s *t* test.

Free Dina (100 µM) and EV-T-Dina (20 µM) were incubated at 37 °C for 0–150 min, respectively, to test the stability of Dina in vitro. As shown in Figure 2a, the concentration of free Dina was reduced by ~50% after 45 min of incubation. By contrast, over 80% of EV-T-Dina remained intact at 150 min post incubation in 37 °C. This suggests that Dina’s encapsulation into EVs significantly enhances its in vitro stability.

A drug release test was performed to characterize the EV-encapsulated Dina in vitro. As shown in Figure 2b, the free Dina was released cumulatively up to 98.82 ± 1.12% after 36 h of dialysis. The EV-T-Dina, by contrast, was released cumulatively to only 12.72 ± 1.55% after 36 h, indicating a significantly reduced diffusion rate of the encapsulated Dina across the dialysis membrane and thus its improved retention due to EV’s molecular weight cut-off size property.

In order to determine the in vivo stability and pharmacokinetics of Dina, two groups of 6-week-old female BALB/c nude mice were injected intraperitoneally with free Dina at the dose of 100 mg/kg or EV-T-Dina at the dose of 100 mg/kg (corresponding to encapsulated Dina 8.24 mg/kg), respectively. After 0.5–8.0 h of injection, the blood samples were collected, and the concentration of serum Dina was determined by HPLC. As shown in Figure 2c, the concentration of serum Dina reached the highest peak (0.2 µg/mL) at 1 h post injection into the free Dina group. By contrast, the EV-T-Dina group showed a significantly higher serum peak Dina concentration of 1.0 µg /mL at 1 h post injection, which remained at 0.6 µg/mL after 8 h of injection. Therefore, the encapsulation of Dina in EVs significantly increases Dina retention time in vivo and greatly improves its pharmacokinetics.

### 3.3. EV-T-Dina Showed Strikingly Enhanced Cytotoxicity and Apoptosis-Inducing Activity in Cancer Cells

**Figure 3:**
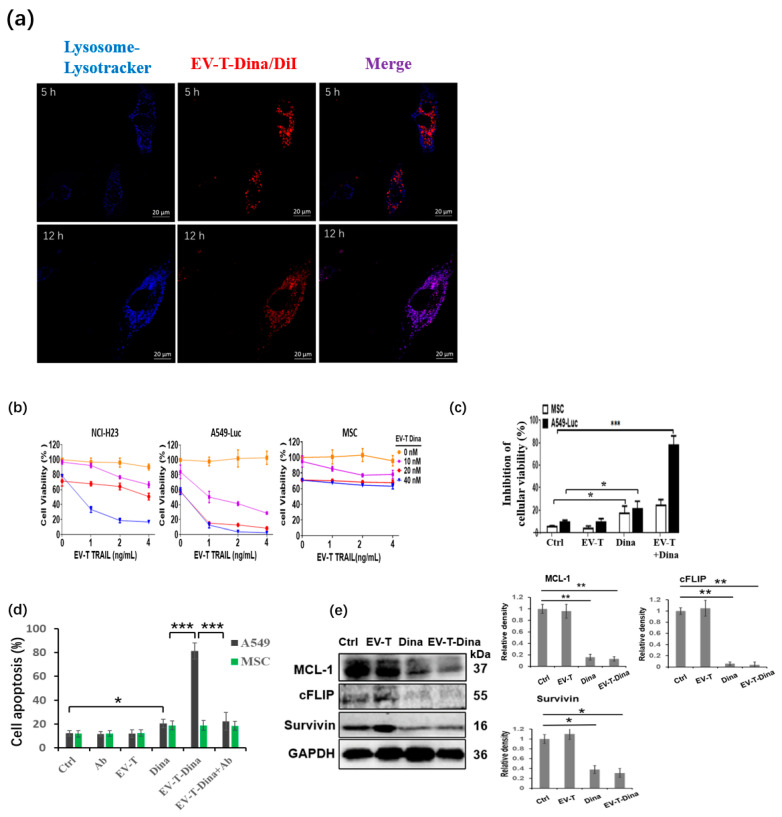
EV-T-Dina inhibits cellular viability and drastically induces enhanced apoptosis in cancer cells. (**a**) Examination of co-localization of cellularly up-taken DiI-labeled EV-T-Dina (EV-T-Dina/DiI) with cellular lysosomes in A549 cells. Cells were seeded and allowed to settle down for 24 h first, and then were co-incubated with EV-T-Dina/DiI for cellular uptake assay for 5 h and 12 h; cellular lysosomes were stained with the lysosome-specific dye (Lysotracker, Yeasen Biotech Co., Ltd., Shanghai, China); Scale bar = 20 μm. (**b**) Assessment of cellular viability and proliferation by using the cell counting kit-8 (CCK-8). Two highly TRAIL-resistant NSCLC lines (NCI-H23 and the luciferase-expressing A549, A549-Luc) and mesenchymal stem cells (MSCs) were treated by EV-T-Dina at indicated combination concentrations of EV-T TRAIL and Dina for 24 h before assessment, respectively. (**c**) Comparison of cytotoxicity of EV-T and Dina, alone or in combination against MSCs and A549 cells. Cells were treated by vehicle (Ctrl), 2 ng/mL EV-T TRAIL (EV-T), 20 nM Dina (Dina), and EV-T (2 ng/mL TRAIL) + Dina (20 nM) for 24 h, followed by cellular viability assay. (**d**) Evaluation of apoptosis in A549-Luc and MSC cells using AF488-Annexin V/PI staining combined with flow cytometry analysis. Cells were treated with PBS vehicle (Ctrl), a human TRAIL-neutralizing antibody (Ab) (100 ng/mL, T3067, Sigma-Aldrich, Shanghai, China), EV-T (2 ng/mL TRAIL), Dina (20 nM), EV-T-Dina (2 ng/mL TRAIL and 20 nM encapsulated Dina), and EV-T-Dina plus Ab for 24 h before evaluation. (**e**) Expressional assessment of anti-apoptotic factors cFLIP, MCL-1, and Survivin by Western blotting in A549-Luc cells treated by vehicle control, EV-T, Dina, and EV-T-Dina with doses described in (**d**), respectively. Uncropped blots are available in Figure S3. All values are mean ± S.E.M (*n* = 3). * *p* < 0.05, ** *p* < 0.01, *** *p* < 0.001, by one-way ANOVA/Bonferroni multiple-comparison post hoc test.

An analysis of the cellular uptake of the EVs was performed in A549 cells to examine the plausibility of using EVs for drug delivery. As shown in Figure 3a, DiI-labeled EV-T-Dina (EV-T-Dina/DiI, red) was readily endocytosed and observed in the cytoplasm of the A549 cells after a 5-h co-incubation. However, after 5 h, the internalized EVs were separated with cellular lysosomes that were labeled blue by the lysosome-specific probe Lysotracker. Interestingly, after a 12-h co-incubation, all the internalized EV-T-Dina/DiIs were observed to be fusing, co-localizing with lysosomes and being lysed, suggesting the subsequent release of encapsulated Dina in cancer cells. This observation demonstrates the suitability of using EVs to deliver drugs for cancer therapy.

Next, the cytotoxicity of EV-T-Dina was examined in two highly TRAIL-resistant NSCLC lines (NCI-H23 and A549) and the normal MSC cells. Low doses of TRAIL from EV-T (0–4.0 ng/mL) and low doses of Dina encapsulated in EV-T (0–40 nM) were combined to perform a cell treatment for 24 h, followed by cell proliferation and viability assays. As shown in Figure 3b, EV-T-Dina demonstrated high cytotoxicity to both NCI-H23 and A549 lines in a dose-dependent manner. The encapsulation of 40 nM Dina in 32 µg/mL EV-T (equivalently 4.0 ng/mL TRAIL) (EV-T-Dina) showed 83.2 ± 6.1% and 98.1 ± 1.8% inhibition rates of cellular viability on NCI-H23 and A549 cells, respectively. The combined treatment, by contrast, showed only a minimal effect on MSCs, although a minor reduction of cellular viability was caused by Dina (Figure 3b), indicating that Dina is toxic to normal cells and thus must be kept in a minimal dosage for therapeutic application. Additionally, a comparison study was performed to further assess and compare the cytotoxicity of EV-T and Dina, alone or in combination on MSC and A549 cells. The obtained results confirmed the sensitizing effect of Dina on EV-T responses in cancerous but not normal cells (Figure 3c).

According to the cytotoxicity results, 2 ng/mL EV-T TRAIL and 20 nM Dina were determined as optimal drug combination doses for an apoptosis assay in both A549 and MSC cells to further evaluate the anticancer activity of EV-T-Dina. As shown in Figure 3d, the Dina alone treatment at 20 nM slightly induced apoptosis in both A549 and MSC cells, but a low dose of EV-T (2 ng/mL TRAIL) alone showed no significant effects. The EV-T-Dina treatment, by contrast, stimulated strikingly augmented apoptosis in A549 cells but not in MSCs in comparison with the Dina alone treatment. Of note, the enhanced apoptosis was abolished by adding a TRAIL-neutralization antibody (T3067), indicating that the synergistic apoptosis induction was due to the combination of Dina and TRAIL in the EVs but not by the co-action of Dina with other EV components. 

cFLIP, MCL-1, and Survivin are three well-known intrinsic inhibitory factors in the TRAIL signaling pathway [26]. The protein expression of the three factors was examined in A549-Luc cells by immunoblotting to reveal any possible molecular mechanisms underlying the synergistic apoptosis induction by EV-T-Dina. As shown in Figure 3e, both Dina (20 nM) alone or its combination with EV-T (2 ng/mL TRAIL) significantly downregulated the expression of cFLIP, MCL-1, and Survivin when compared with the PBS vehicle control treatment. This explains the strikingly augmented apoptosis induction in the A549-Luc cells by EV-T-Dina.

Collectively, these data demonstrate that EV-T-Dina is highly efficient for inducing apoptosis in the TRAIL-resistant A549 line with a mechanism associated with the concomitant downregulation of cFLIP, MCL-1, and Survivin.

### 3.4. Preparation of Aerosolized Therapeutics

Therapeutic solutions were converted into aerosols by using a portable pressurized nebulizer. The appropriate insert was chosen for the nebulizer, aiming to mainly produce an aerosol particle size below 5.0 µm in diameter. This size was chosen to ensure that the therapeutic aerosols could be inhaled into the bronchioles and alveoli.

**Figure 4:**
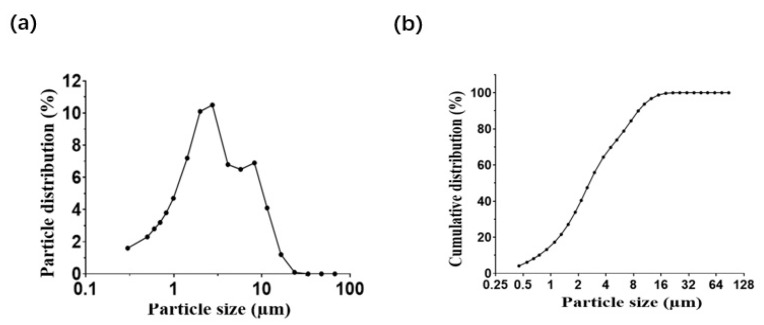
Size distribution of aerosolized drug droplets. (**a**) Examination of size distribution of EV-T-Dina aerosol by the laser diffraction system (LDS); (**b**) Cumulative size distribution of aerosolized therapeutics.

A laser diffraction system was used to measure the size distribution of aerosol particles and droplets. As shown in Figure 4a, the mean median diameter (MMD) of the prepared aerosol particles is 2.65 ± 0.14µm, and the cumulative size distribution reaches 81.12 ± 2.51% for particles below 5.0 µm (Figure 4b). These results indicate that the prepared aerosols are suitable for the inhalational treatment of lung cancer.

### 3.5. EV Improved Pulmonary Delivery of Aerosolized Therapeutics

**Figure 5:**
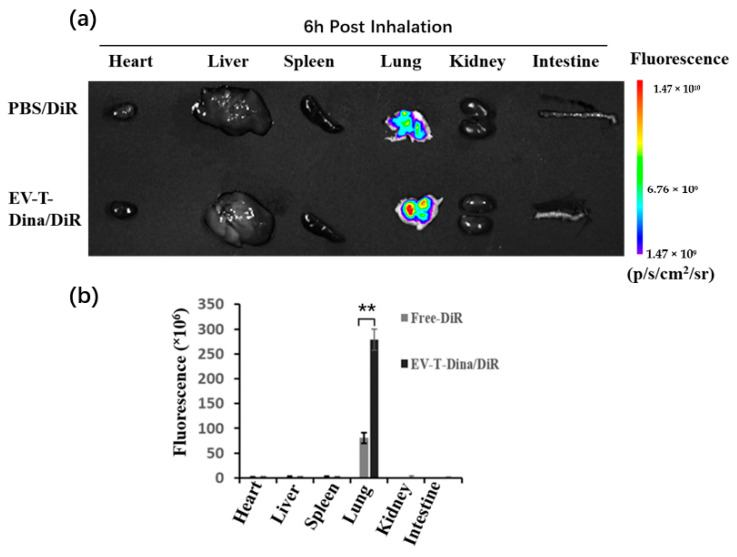
Unique distribution of inhaled EV-T-Dina/DiR in lungs of mice. (**a**) Bio-distributional observation of aerosolized free DiR (Free-DiR) or EV-T-Dina labeled by DiR (EV-T-Dina/DiR) in nude mice by an in vivo imaging system (IVIS). Free-DiR and EV-T-Dina/DiR were first aerosolized and inhaled by animals, and then examined for DiR biodistribution in isolated animal organs including heart, liver, spleen, lung, kidney, and intestine 6 h post inhalation by IVIS. (**b**) Quantification and comparison of DiR fluorescence in organs by IVIS. All values are means ± SD (*n* = 4), ** *p*< 0.01 versus free-DiR group, by Student’s *t*-test.

Free DiR and DiR-labelled EV-T-Dina (EV-T-Dina/DiR) were aerosolized and inhaled by the mice to examine their in vivo biodistributions. The mice were sacrificed 6 h post inhalation, and their organs were excised and examined to determine their DiR distribution. As shown in Figure 5a,b, a DiR fluorescence signal was uniquely seen to be located in the lung but not in other organs such as the heart, liver, spleen, kidney, and intestine for both free DiR- and EV-T-Dina/DiR inhalation. Of note, significantly more EV-T-Dina/DiR were observed to accumulate in the lung than free DiR, suggesting the better accumulation/retention of DiR delivered by EVs in lung tissues. This observation demonstrates that EVs can be harnessed to improve the pulmonary delivery of therapeutics.

### 3.6. EV-T-Dina Inhalation Therapy Drastically Suppressed the Development of Established Orthotopic Lung Tumors

**Figure 6:**
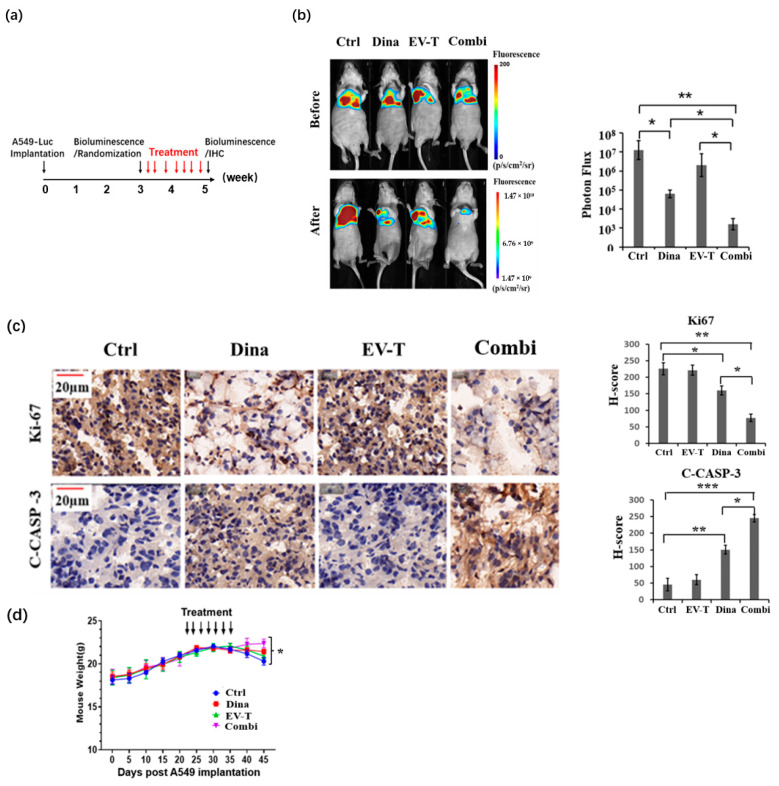
Inhalation therapy with aerosolized EV-T-Dina strikingly suppressed the development of established orthotopic lung tumors in nude mice. (**a**) In vivo experimental schedule is shown. (**b**) Tumor growth was assessed 3 days post last inhalation treatment by bioluminescence imaging (Photon Flux, MILabs, Perkin Elmer, Shanghai, China). Four inhalation treatment groups were tested including vehicle control (2 mL/mouse, Ctrl), 160 µg EV-T (~20 ng TRAIL equivalently/2 mL/mouse), Dina (20 µg/2 mL/mouse), and ~180 µg EV-T-Dina (~20 ng TRAIL by 160 µg EV-T + 20 µg encapsulated Dina/2 mL/mouse, Combi). In total, seven treatments were given to each animal with a 2-day interval. Values are means ± SD (*n* = 5). One representative mouse in each group is shown. (**c**) Immunohistochemistry analyses on Ki67 and cleaved caspase-3 (C-CASP-3) in lung tumor sections of treated mice, respectively, scale bar: 20 μm. (**d**) Curves of animal body weight post A549-Luc cell implantation and along with the inhalation therapies. Values are means ± SD. (*n* = 5). * *p* < 0.05; ** *p* < 0.01, *** *p* < 0.001; by one-way ANOVA/Bonferroni multiple-comparison post hoc test.

Having seen the synergistic apoptosis induction in the A549 cells by EV-T-Dina and established the unique distribution of the inhaled EV-T-Dina aerosol in the lungs, the therapeutic potency of the aerosol was next evaluated in an orthotopic lung tumor model in vivo, as shown in Figure 6a.

Luciferase-expressing A549 cells (A549-Luc) were implanted into the lungs of nude mice to induce lung tumor development. Three weeks post implantation, the mice were examined for luciferase bioluminescence, followed by randomization to assign animal treatment groups with comparable bioluminescence and thus similar initial tumor burdens in test groups. Four inhalation treatment groups were tested, including vehicle control, EV-T, Dina, and EV-T-Dina and seven treatments were performed in total for each mouse using a pressurized nebulizer in a 2-week treatment cycle. The treatment results were determined by bioluminescence imaging in vivo and are shown in Figure 6b. While EV-T inhalation alone marginally ameliorated lung tumor development when compared with the control, both Dina inhalation alone and EV-T-Dina therapy showed significant inhibitory effects towards tumor growth. However, EV-T-Dina inhalation appeared significantly more effective than that of Dina alone.

The animals’ lung tumors were subsequently removed and assessed for the cell proliferation marker ki67 and cleaved (activated) caspase-3 expression by IHC staining at the experimental end point. As shown in Figure 6c, both inhalation therapies using Dina or EV-T-Dina significantly decreased Ki67 expression in tumor cells compared with the vehicle PBS control, indicating the suppression of tumor growth and development. In addition, both therapies also caused the activation of caspase-3, suggesting the significant induction of apoptosis in these cancers. However, EV-T-Dina treatment showed further improved efficacy than Dina therapy alone in the cancer cells. Additionally, the EV-T-Dina treatment group of mice showed a steady weight gain (Figure 6d), indicating the good tolerance of the therapy. All the other groups, by contrast, showed a slight decline in their body weights at the last test week, likely due to the burden of the lung tumors.

Moreover, mouse organs including heart, liver, spleen, and kidney were collected from both the vehicle and EV-T-Dina inhalation groups at the experimental endpoint and evaluated for apoptosis by TUNEL staining (Figure S1). This was conducted to assess the safety of the therapy. There was no significant apoptosis induction in any of the examined animal organs from the EV-T-Dina group compared with the vehicle control. This data suggests the satisfactory safety of the EV-T-Dina inhalation therapy.

Collectively, these observations demonstrate that the inhalation of EV-T-Dina aerosol constitutes a novel therapy, which is highly effective and safe for lung cancer treatment.

## 4. Discussion

In this study we have shown: (1) that Dina can be encapsulated into TRAIL-expressing EVs to produce the complexed agent EV-T-Dina, (2) that integrated Dina and TRAIL are synergistic for the specific killing of cancer cells with high efficiency, (3) that EV encapsulation improves Dina stability both in vitro and in vivo, (4) that EV-mediated aerosol delivery showed both a unique distribution and an improved drug retention in the lungs, (5) and that the aerosol delivery of EV-T-Dina demonstrated high efficacy and good safety for lung cancer treatment.

Accounting for about 85% of all types of lung cancer, NSCLC are generally treated by surgical resection in combination with systemic chemotherapy either preoperatively or postoperatively. Although intending to find a cure, the therapeutic efficacy of such a treatment strategy is frequently affected by chemotherapy resistance in NSCLC. EGFR-targeting agents are primarily used as first-line drugs for the treatment of EGFR-mutant NSCLC, such as erlotinib, gefitinib, and afatinib. These drugs are beneficial for extending the life spans of patients but possess limited effectiveness due to drug resistance. Moreover, these first-line treatments are often associated with adverse side effects, such as an elevation in serum aminotransferase levels during therapy, indicating apparent liver toxicity.

As a second generation of CDK inhibitor and an experimental drug, Dina (SCH-727965) has been widely tested in phase II clinical trials for treating advanced breast cancer [27], NSCLC [28], multiple myeloma [29], and advanced melanoma [29], and has been tested in a phase III trial for treating relapsed/refractory chronic lymphocytic leukemia (CLL) [30]. These trials demonstrated some antitumor activity from Dina but the efficacies were not superior to the current first line of drugs. While these trials generally exhibited acceptable safety and tolerability, some grade 3 or 4 treatment-related adverse effects were seen, such as leukopenia, neutropenia, febrile neutropenia, and elevated aspartate aminotransferase. Therefore, other strategies must be combined with Dina treatment to potentiate its therapeutic efficacy and reduce its adverse side effects.

The pulmonary delivery of therapeutics has been employed to treat all types of lung diseases [11] including chronic obstructive pulmonary disease (COPD) [31], asthma [32], emphysema [11], bronchitis [33], tuberculosis [34], and lung cancer [35]. Aerosol inhalation is a noninvasive approach to drug delivery to the lungs. However, the performance of aerosol therapy can be affected by several key aspects, including the drug formulation, drug delivery vector, particle size, inhalation device, and patient selection. In addition, drug solubility plays an essential role in determining the therapy efficacy, since drugs with low solubility tend to precipitate or crystalize, causing nebulization difficulties.

EVs are an emerging novel drug delivery system with many advantages [36], such as a versatile loading capacity to accommodate both soluble and insoluble cargoes, active trans-endothelial transport [37], good stability in vivo, flexibility for modification, tumor tropism, immune evasion, and satisfactory safety. Dina is insoluble in water, and DMSO or ethanol must be used to improve its solubility. In addition, Dina solution is not stable, particularly at 37 °C as shown in this study. However, the encapsulation of Dina in EVs not only surmounts the solubility obstacle but also improves its stability both in vitro and in vivo, facilitating the preparation of aerosolized agents.

The observed low stability of free Dina solution at 37 °C may provide evidence supporting the previously examined short plasma half-life of Dina in mice [38], given the fact that the internal body temperature is around 37 °C. Interestingly, EV-encapsulated Dina appeared significantly more stable at 37 °C both in vitro and in vivo. The underlying mechanism remains to be elucidated. However, one possible reason is the expression of the “self” protein CD47 on MSC-derived EVs [39,40], which protects EV-T-Dina from phagocytic clearance and thus prolongs the circulation and retention of the encapsulated Dina in blood. By contrast, the infused free Dina is more susceptible to phagocytic clearance without the isolation effect of CD47.

Chemotherapy is a common cancer treatment approach with some inevitable shortcomings, such as low bioavailability, poor tumor tropism, a short plasma half-life, and systemic adverse side events, frequently resulting in the onset of drug resistance in tumors [41]. As shown in this study, EVs, as a natural nanoparticle vehicle for drug delivery, can be harnessed to improve the formulation of therapeutics. They are especially suitable for the co-loading of both hydrophobic and hydrophilic drugs for combined therapies due to their phospholipid membrane and aqueous core structure [13]. The EV-based combinatory therapy may facilitate tumor-targeted drug delivery, improve therapeutic efficacy, reduce drug doses, and thus enhance the treatment safety as well [42].

Previously, we found that TRAIL-expressing EVs (EV-T) can be combined with Dina to produce synergistic anti-tumor activity [18]. Dina alone could induce apoptosis to some extent in cancer cells [27,43], which is likely associated with the concomitant downregulation of MCL1, Survivin, and cFLIP in cells. TRAIL is a pro-apoptotic factor, which functions to trigger an extrinsic apoptosis signaling pathway [44]. However, this apoptosis signaling pathway is generally blocked by cellular antiapoptotic factors including MCL1, Survivin, and cFLIP [45,46]. Thus, Dina is used to greatly amplify and augment the apoptosis signaling stimulated by TRAIL due to its activities towards suppressing the expression of antiapoptotic factors. This may explain the synergistic apoptosis induction effect between Dina and EV-T in cancer cells.

In this study, we further demonstrated that Dina and TRAIL can be integrated into EVs to produce the complexed agent EV-T-Dina, which is highly effective towards apoptosis induction in cancer lines. More importantly, EV-T-Dina can be delivered into lungs by aerosol inhalation, demonstrating a unique lung distribution and good safety. This strategy has several advantages, such as the lung enrichment of EV aerosols, the synergistic action of the combined drugs, the improved solubility and stability of Dina, and an improved therapeutic efficacy and safety. This can be an example to be used towards the pulmonary route delivery of other therapeutic agents alone or in combination. To the best of our knowledge, this is the first time that the EV-based aerosol has been prepared and tested for the treatment of lung cancer.

**Figure 7:**
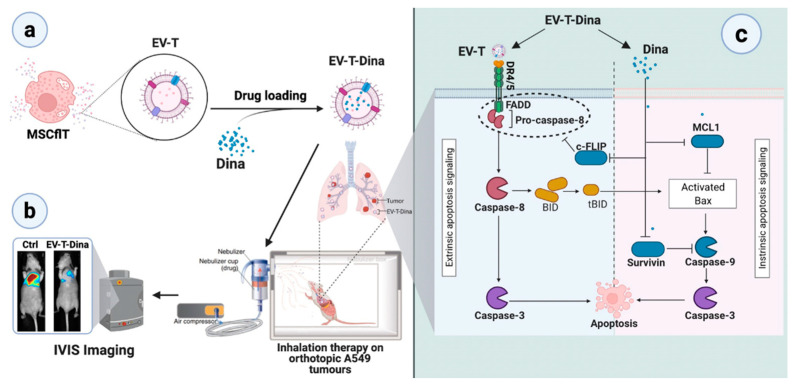
Schematic overview of inhalational therapy on orthotopic A549 lung tumors by the complexed EV-T-Dina agent. (**a**) TRAIL-expressing MSCs (MSCflT) were used to produce TRAIL-loaded extracellular vesicles (EV-Ts), which can be treated by sonication for Dina encapsulation to prepare the complexed therapeutic agent EV-T-Dina; (**b**) The EV-T-Dina solution was nebulized to prepare aerosol therapeutics for the inhalational treatment of orthotopic A549-Luc lung tumors in mice; (**c**) The pulmonary co-delivery of TRAIL and Dina showed synergistic apoptosis-inducing activity in A549 tumors via concomitant suppression of the antiapoptotic factors cFLIP, MCL-1, and Survivin both in vitro and in vivo.

In this study, Dina, after its encapsulation in EV-T, was administered through inhalation at a low dosage and was shown to drastically induce augmented apoptosis in A549 lung cancers without any observed adverse side events, suggesting the plausibility of using the EV-based aerosol delivery of combined therapeutics for better efficacy and safety (Figure 7). In the future, it would be worthwhile to attempt the aerosol delivery of combined EV-T and the first-line lung cancer drugs for the potential improvement of both therapeutic efficacy and biosafety. Additionally, other lung cancer treatment strategies may be combined in EVs to develop novel aerosol therapies, such as immunotherapy, gene therapy, siRNAs, and oncolytic viruses.

This study has demonstrated the plausibility of developing a human lung cancer inhalation therapy based on drug delivery by EVs. However, some concerns remain to be clarified. First, the therapeutic efficacy revealed in an experimental lung cancer model may not guarantee a comparable effectiveness in human diseases. Second, the therapeutic safety for human lung cancer needs to be clarified, although a promising treatment tolerability has been observed in this study. Third, the combination of TRAIL and Dina was only tested for being highly effective for the specific killing of two lung cancer lines, H23 and A549; next, the effectiveness of this combined therapy needs to be extended to a broader range of lung cancer lines. Fourth, a patient selection criterion may need to be established for the precision application of the therapy. Therefore, what we have surely demonstrated now is the plausibility of using EVs for the lung tissue-targeted delivery of therapeutics. Importantly, this study rationalizes further investigations of the EV-mediated pulmonary delivery of various therapeutic agents, alone or in combination, for the treatment of lung diseases.

## 5. Conclusions

In conclusion, chemotherapies such as Dina can be encapsulated in genetically engineered anti-cancer EVs, such as EV-T, to produce complexed inhalation agents such as EV-T-Dina, which concomitantly suppress the expression of innate apoptosis inhibitors such as cFLIP, MCL-1, and Survivin in cancer cells, thereby improving the therapeutic potential and treatment safety of lung cancer therapeutics.

## Data Availability

The data presented in this study are available in the article and Supplementary Materials.

## Supplementary Materials

The following supporting information can be downloaded at: https://www.mdpi.com/article/10.3390/cancers14143550/s1, Figure S1: TUNEL analysis of animal organs by immunohistochemistry (IHC); Figure S2: Full blots for Figure 1e; Figure S3: Full blots for Figure 3e.
