# Supplementary material for: Pulmonary Delivery of Extracellular Vesicle-Encapsulated Dinaciclib as an Effective Lung Cancer Therapy
Source: Cancers (Basel). 2022 Jul 21;14(14):3550. doi: 10.3390/cancers14143550 (PMC9318050; doi:10.3390/cancers14143550)
Supplement: Supplementary file 1 [file cancers-14-03550-s001.zip › cancers-1810180-supplementary.pdf]

Supplementary Material: Pulmonary Delivery of Extracellular Vesicle-Encapsulated Dinaciclib as an Effective Lung Cancer Therapy

Qian Yuan, Kui Su, Shuyi Li, Xinyi Long, Lang Liu, Minghui Yang, Xin Yuan, Jianwu Sun, Junhua Hu, Qin Li, Yu Zhao and Zhengqiang Yuan

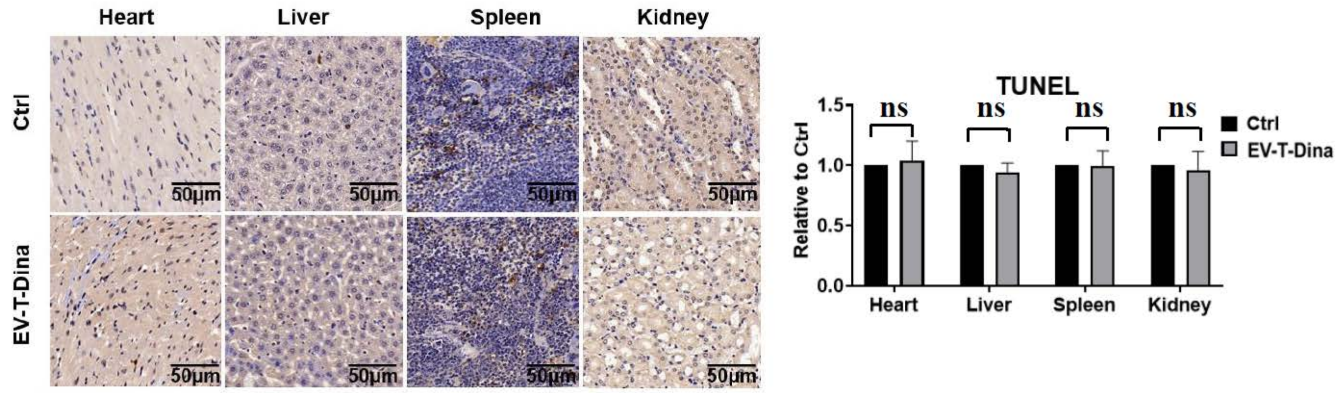

Figure S1. TUNEL analysis of animal organs by immunohistochemistry (IHC). Animal organs includign heart, liver, spleen, and kidney were collected from mice treated by inhalation of vehicle (Ctrl) or EV-T-Dina and analysed for apoptosis by TUNEL staining at the experimental endpoint. Eight random microscopic fields were selected for positive labeling quantification of each sample by using the Image-pro plus 6.0 software. The quantification of positive labeling signal intensity is shown relative to control for which the value was set as 1.0. All values are presented as means ± SD (n = 8), ns = not significant, by Student's t test.

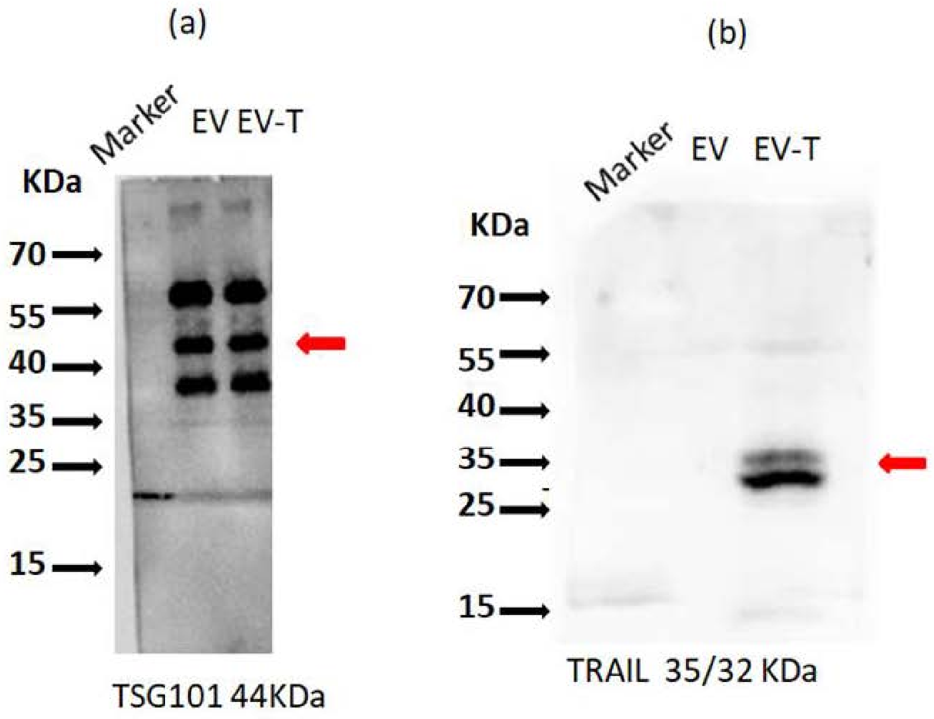

Figure S2. Full blots for Figure 1e. Whole immunoblotting for expression of TSG101 (**a**) and TRAIL (**b**) with molecular weight ladder showing bands from 15 kDa to 70 kDa on the left.

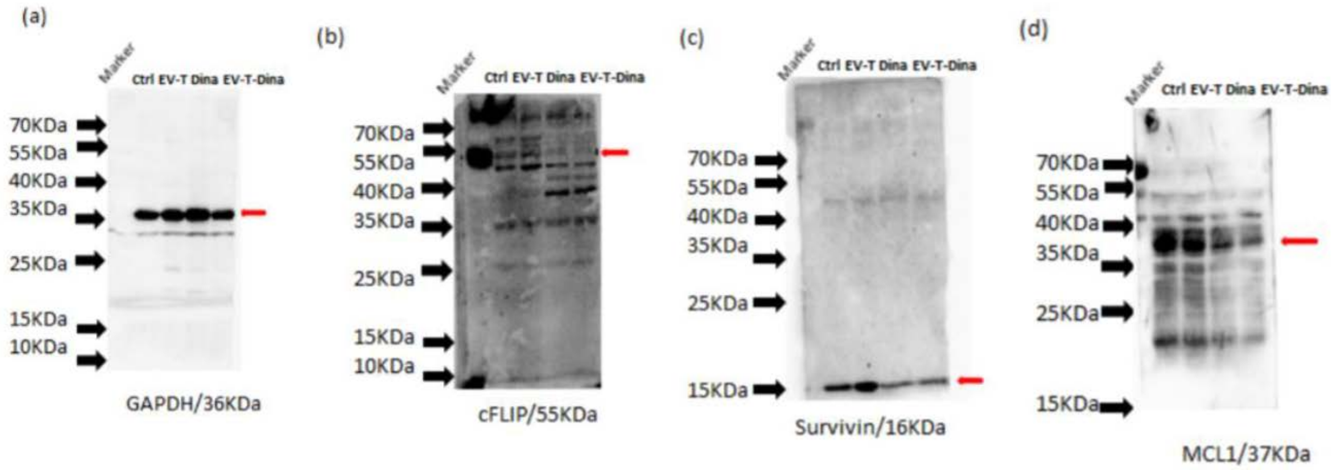

Figure S3. Full blots for Figure 3e. Whole immunoblotting for expression of GAPDH, cFLIP, Survivin and MCL-1 with molecular weight ladder showing bands from 15 kDa to 70 kDa on the left. The sampling order from left to right is: Marker, Vehicle (Ctrl), 2.0ng/mL of EV-T (EV-T), 20 nM of Dinaciclib (Dina), and the combination of same doses of TRAIL and Dina in EVs (EV-T-Dina).
